# Controllable design of super-oscillatory lenses with multiple sub-diffraction-limit foci

**DOI:** 10.1038/s41598-017-01492-y

**Published:** 2017-05-02

**Authors:** Muyuan Li, Wenli Li, Haoyong Li, Yechuan Zhu, Yiting Yu

**Affiliations:** 1 Key Laboratory of Micro/Nano Systems for Aerospace (Ministry of Education), Northwestern Polytechnical University, Xi’an, 710072 China; 2 Key Laboratory of Micro- and Nano Electro-Mechanical Systems of Shaanxi Province, Northwestern Polytechnical University, Xi’an, 710072 China

## Abstract

The conventional multifocal optical elements cannot precisely control the focal number, spot size, as well as the energy distribution in between. Here, the binary amplitude-type super-oscillatory lens (SOL) is utilized, and a robust and universal optimization method based on the vectorial angular spectrum (VAS) theory and the genetic algorithm (GA) is proposed, aiming to achieve the required focusing performance with arbitrary number of foci in preset energy distribution. Several typical designs of multifocal SOLs are demonstrated. Verified by the finite-difference time-domain (FDTD) numerical simulation, the designed multifocal SOLs agree well with the specific requirements. Moreover, the full-width at half-maximum (FWHM) of the achieved focal spots is close to *λ/*3 for all the cases (*λ* being the operating wavelength), which successfully breaks the diffraction limit. In addition, the designed SOLs are partially insensitive to the incident polarization state, functioning very well for both the linear polarization and circular polarization. The optimization method presented provides a useful design strategy for realizing a multiple sub-diffraction-limit foci field of SOLs. This research can find its potentials in such fields as parallel particle trapping and high-resolution microscopy imaging.

## Introduction

Multifocal optical elements are important for such applications as parallel particle trapping and three-dimensional imaging system^1–4^. For the purpose, much research has been reported both theoretically and experimentally on the realization of focusing the light into several focal spots simultaneously. In most recent studies, multiple foci are mainly obtained by employing two different methods. Through modulating the incident cylindrical vector beam in a 4Pi focusing system, several equidistant multiple spots can be generated along the optical axis^1, 2^. Furthermore, the metalens with longitudinal multiple foci has also been proposed^5, 6^. However, these multifocal optical elements cannot precisely control the energy distribution among the realized focal spots, neither the relative positions nor the actual sizes. Our goal is to design the high-quality multifocal lenses owning the required number of foci and the preset energy distribution among them.

On the other hand, resolving power is restricted by the Rayleigh diffraction limit 0.61λ/NA (where NA is numerical aperture) for an ideal optical system^7^. Overcoming this resolution barrier can improve the imaging quality, or greatly decrease the size of a single particle that can be manipulated. In recent years, much attention has been paid to utilize the super-oscillation theory^8, 9^ to design the planar metallic lenses composed of an array of circular nanorings with different widths^10–13^, and the sub-diffraction-limit focusing performance has been successfully realized^12, 14–16^. The achieved lenses are thus named as the super-oscillatory lenses (SOLs). They can create a sub-diffraction-limit hotspot at a distance far beyond the near-field region, thus without the contribution of evanescent waves. In 2012, N. I. Zheludev *et al*. reported an optical microscope showing an imaging resolution close to *λ*/6 by using a SOL for directly focusing the laser light into a subwavelength spot over more than 10 µm away and by precisely tailoring the interference of a large number of beams diffracted from a nanostructured binary amplitude-type mask^17^. Therefore, the design of nanostructured mask plays a paramount role in developing the SOLs for practical applications. According to the available publications, the design theories for a far-field superfocusing SOL have been based on the scalar angular spectrum theory^10, 17^, vectorial angular spectrum (VAS) theory^13, 14, 18^, or vectorial Rayleigh-Sommerfeld diffraction integral^15^. However, it is difficult to achieve a precisely controllable high-quality light field with these ordinary methods.

In this paper, we suggest a multi-objective and multi-constraint optimization model, aiming to implement the SOLs with an arbitrary number of sub-diffraction-limit focal spots along the optical axis and the desired energy distribution between them. The optimizing procedure of the model is designed adopting the Matlab programming language based on the genetic algorithm (GA) and the fast Hankel transform algorithm. It can flexibly control the intensity of electric field immediately behind the lens, making the number of foci arranged, as well as their relative positions and sizes. The achieved SOLs based on the presented method are verified by the rigorous electromagnetic simulation using the finite-difference time-domain (FDTD) numerical computation. Although the design described mainly suits for the linearly polarized beam (LPB), it is also applicable for the other polarized waves, like the circularly, radially, and azimuthally polarized beams.

## Design and optimization procedure

### Integral representations

**Figure 1:**
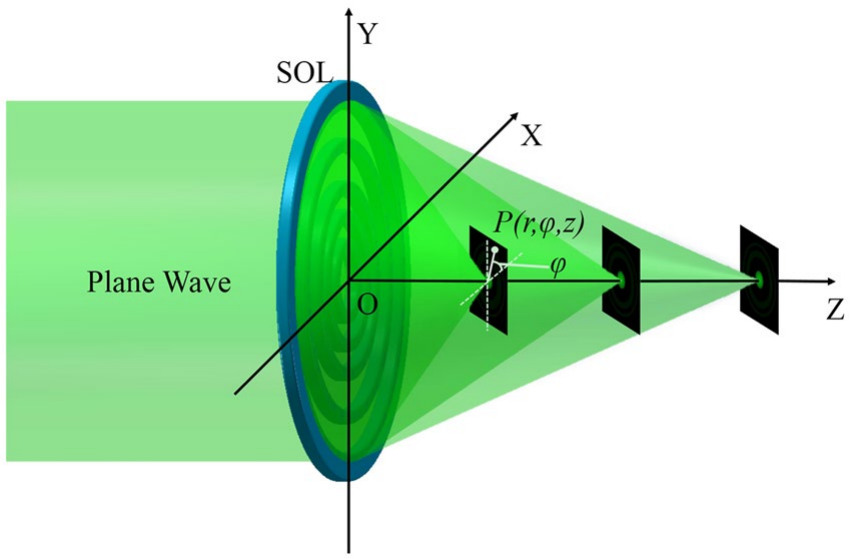
Schematic diagram of multiple sub-diffraction-limit focusing by a SOL.

The subwavelength optical field pattern can be constructed by employing the SOL consisting of multiple concentric nanorings through the interference of a massive transmitted diffraction beams. Assuming the LPB (electric field polarized along the *X* direction) illuminates normally on the SOL and propagates along the +*Z* direction, as shown in Fig. 1, according to the VAS theory under the cylindrical coordinate system, the electric field components of an arbitrary point *P*(*r*, *φ*, *z*) on the observation plane (*Z* > 0) can be expressed as^14, 19, 20^

1 $$\{\begin{array}{c}{E}_{x}(r,z)={\int }_{0}^{\infty }{A}_{0}(l)\exp [j2\pi q(l)z]{J}_{0}(2\pi lr)2\pi l{\rm{d}}l\\ {E}_{y}(r,z)=0\\ {E}_{z}(r,\phi ,z)=-j\,\cos \,\phi {\int }_{0}^{\infty }\frac{l}{q(l)}{A}_{0}(l)\exp [j2\pi q(l)z]{J}_{1}(2\pi lr)2\pi l{\rm{d}}l\end{array}$$

where *q(l)* is the longitudinally spatial frequency component and *A*_0_(*l*) is the angular spectrum of the electric field in the mask; *J*_*n*_(·) denotes the *n* ^*th*^-order Bessel function^19^. The transversely and longitudinally polarized electric energy densities are calculated by ${|{E}_{r}(r,z)|}^{2}={|{E}_{x}(r,z)|}^{2}$ and ${|{E}_{z}(r,\phi ,{\rm{z}})|}^{2}$, respectively; thus, the total electric energy density is $I(r,\phi ,z)={|{E}_{x}(r,z)|}^{2}+{|{E}_{z}(r,\phi ,z)|}^{2}$. If SOLs are illuminated by circularly polarized beam (CPB), the total electric energy density can be obtained from two orthogonally polarized LPBs, referring to^20^ $I(r,\phi ,z)=2({|{E}_{x}(r,z)|}^{2}+{|{E}_{z}(r,\phi ,z)|}^{2})$, where *E*_*x*_ and *E*_*z*_ are described in Equation (1). In a high numerical aperture (NA) microscopic imaging system, the transversely polarized electric-field component is dominant, while the longitudinal one is always strongly attenuated in the image plane due to the polarization filtering of this imaging system^21, 22^. Therefore, we do not consider the longitudinal component and the total electric energy density profiles of LPB and CPB tend out to be almost the same, quantized as $I(r,z)={|{E}_{x}(r,z)|}^{2}$ and $I(r,z)=2{|{E}_{x}(r,z)|}^{2}$, respectively. What mentioned above demonstrates an acceptable agreement with the experimental results^12, 14, 15, 17^.

### Optimization model

**Figure 2:**
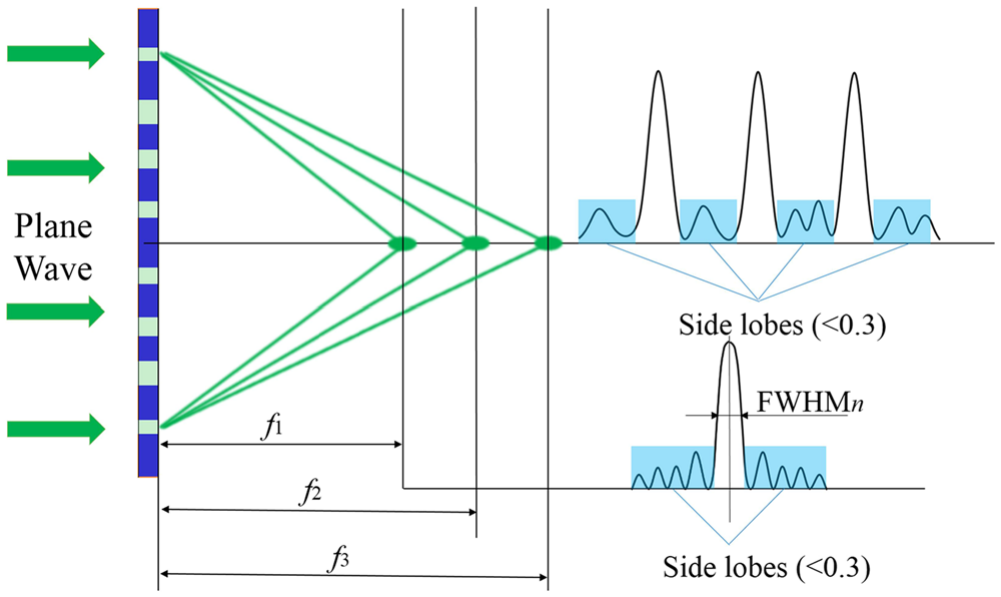
The strategy to construct the multifocal light field.

Figure 2 presents the schematic of a multifocal SOL where a metallic film is etched with a great number of nanorings of specially designed widths. Taking LPB as an example for clarity, we consider the total electric energy density is approximated by $I(r,z)={|{E}_{x}(r,z)|}^{2}$. The constraint model of GA can be optimized with the required optimization targets using the three-dimensional (3D) intensity distribution *I*. We constrain *I* along two orthogonal directions, including the optical axis and transverse axes in every focal plane. Then, a constrained linear programming model is extracted from an optimized design of multiple foci with optical transmittance as the objective function. In order to achieve a required high-quality sub-diffraction-limit multifocal field, which is influenced by many factors such as the focal length, full-width at half-maximum (FWHM), surrounding side lobes, and light uniformity of these focal spots, as shown in Fig. 2. We employ the Matlab programming language based on the GA to design the binary amplitude-type SOL that implements the predefined axial-intensity modulation over a given region. Here, the model of three objectives and three constraints is established to control the multifocal field’s prescribed parameters. Hence, a constraint optimization model is built up as Equations (2) and (3).

2 $${Minimize}\{\begin{array}{c}{I}_{1m}=\,{\rm{\max }}\{\max [I(0,{f}_{m-};{t}_{i})]\,\max [I(0,{f}_{m+};{t}_{i})]\}\\ {I}_{2m}={\{{\rm{\max }}[I(0,{f}_{1};{t}_{i})-\mu \cdot I(0,{f}_{m};{t}_{i})]\}}^{2}\quad \quad \quad {m}=1,2\mathrm{...}{M}\\ {I}_{3m}=I(\frac{FWHM}{2},{f}_{m};{t}_{i})\end{array}$$

3 $$\begin{array}{c}{Subject}\,{to}\\ \{\begin{array}{c}I(0,{z}_{x};{t}_{i})\le 0.3,{z}_{x}\in (0,{f}_{1}-\frac{{D}_{f}}{2})\cup ({f}_{n}+\frac{{D}_{f}}{2},{f}_{n+1}-\frac{{D}_{f}}{2})\cup ({f}_{M}+\frac{{D}_{f}}{2},z),n=1,2,\ldots ,M-1\\ I(r,{f}_{m};{t}_{i})\le 0.3,\frac{FWHM}{2}\le r\le \kappa \frac{FWHM}{2}\\ {t}_{i}\in \{0,1\}\,i=1,2,\ldots ,N\end{array}\end{array}$$

where the electric-field intensity *I* is normalized; *M* is the number of focal spots. *f*_*1*_, *f*_*2*_ … *f*_*m*_ represent the *m-th* location of the focal length; ${f}_{m-}={f}_{m}-{D}_{f}/2$ and ${f}_{m+}={f}_{m}+{D}_{f}/2$ with *D*_*f*_ being the depth of focus; *t*_*i*_ is the transmittance value of the *i*-th annular ring and *N* is the total number of rings contained in the mask. For the binary amplitude-type annular mask, the contained concentric rings are initially set to be equidistant and each ring can have either unit or zero transmittance, so the binary amplitude transmittance is encoded straightforward using the two digits {0, 1}.

We built the three-objective and three-constraint model to control the main properties of the 3D intensity distribution behind the SOLs. The first objective function *Min*.(*I*_*1m*_) means that the energy surrounding each focal spot along the optical axis should be less than the energy of each focus as much as possible, that is to ensure the intensity of the targeted focal spots are the peaks of the axial intensity distribution, which is useful for controlling the number of focal spots; the second objective function *Min*.(*I*_*2m*_) represents the ratio of focusing intensity; *μ* represents the difference of the intensity distribution between foci; *μ* = 1 means that the intensity distribution between the focal spots changes a little, thus each spot has the similar energy. It’s beneficial to achieve several homogeneous focal spots. The third objective function *Min*.(*I*_*3m*_) controls the sizes of the hotspots in the focal planes as small as possible, aiming to decrease the FWHM.

On the other hand, the three constraints are used to control the light field parameters that we prescribe before optimizing, such as the intensity of the side lobe and FWHM. These objectives are related and difficult to control. Thus, we set a specific fluctuation range of the electric intensity distribution at different locations to ensure the light needed. As shown in Fig. 2, the intensity of the other points on the optical axis is set to below 0.3. A super-oscillation field always exists accompanied by high-energy side lobes, which will impede its widespread application^23^. Therefore, for the focusing planes perpendicular to the optical axis, the normalized intensity within the range of $\frac{FWHM}{2}\le r\le \kappa \frac{FWHM}{2}$, the radial width of the transition dark region between the central main lobe and the surrounding side lobes is supposed to be lower than 30% of the peak intensity of the central lobe. A specific fluctuation range of the side lobe was set to ensure the light field as required. If the parameter is unsuitable, it may be difficult to convergence to an optimal solution. After analyzing the energy relationship between the side lobe and the central spot, a side lobe factor of 0.3 is chosen in our design.

GA is widely used for such problems due to its powerful parallel and global searching capability^24, 25^. Since a sub-diffraction-limit multifocal field is influenced by many factors, and these factors often conflict with each other. When we reduce the spot sizes, there always comes along with the increase of intensity for sidebands. Therefore, there exists a tradeoff of the light energy between the central foci and their side lobes. A feasible tradeoff is achieved when there are no significant side lobes, and meanwhile, the spot sizes maintain a good uniformity beyond the diffraction limit. The multi-objective optimization problem makes the components minimum simultaneously. The problem usually has no unique, perfect solution, but a set of nondominated, alternative solutions, known as the Pareto-optimal set^25^. Multi-objective optimization arises from the need for a strategy to address the multiple design factors for practical problems. As for GA, a fitness function must be set to make the optimized results and the objectives as close as possible. In our design, objective functions 1~3 in Equation (2) are served as the individual fitness functions when the genetic operation, named as “selection”, is performed in GA. Intuitively, these three individual fitness functions can be weighted and summed up to formulate a single-objective optimization. Here, we assign a weighted coefficient *w*_*j*_ to each objective function *I*_*j*_, so that the problem is converted to a single-objective problem with the objective function defined as,

4 $${\rm{\min }}(I)=\,{\rm{\min }}\{{w}_{1}[\sum _{m=1}^{M}{I}_{1m}(r,\phi ;{t}_{i})]+{w}_{2}[\sum _{m=1}^{M}{I}_{2m}(r,\phi ;{t}_{i})]+{w}_{3}[\sum _{m=1}^{M}{I}_{3m}(r,\phi ;{t}_{i})]\}$$

To achieve a compromised high-quality light pattern, the suitable weighted coefficients are important, which are set according to the importance of three objective functions. Here, we presume the weighted coefficients *w*_1_, *w*_2_ and *w*_3_ as 0.4, 0.4 and 0.2, respectively. GA is set to hold a population of 500, with a crossover probability of 0.7, and a mutation probability of 0.007. Through numerical calculations, it is found that the required SOLs can be steadily satisfied after several hundred iterations by using the above configurations. In addition, a fast Hankel transform algorithm can be applied to dramatically accelerate the calculation speed^26^.

## Result and Discussions

In the following examples, an illumination wavelength of 532 nm is used in oil immersion medium (*n* = 1.515). The diameter of the mask is designed to be 20 μm with a total ring number of 100, so the minimum annular width is 100 nm. 200 iterations are sufficient to ensure the convergence and are thus used for each algorithm. SOLs #1~#6 are optimized and listed in Table 1. The proposed scheme has been validated by the 3D FDTD method for a 25 nm-thick aluminum film. According to the optimization procedure, the transmittance functions of SOLs are achieved according to the different requirements, as shown in Table 1. In order to describe the SOL (might contain several hundred rings) more compactly, the transmittance value *t*_*i*_ is encoded from the first ring (innermost) to the *N* ^th^ ring (outermost) by continuously transforming every four successive binary digits into one hexadecimal digit. Taking the SOL #2 as an example, the first hexadecimal digit “A” denotes the real transmittance values of “1010”. “1” and “0” represent the transparent and opaque annulus, respectively.

**Table 1:** Targeted parameters and transmittance functions of the optimized binary amplitude-type SOLs.

	*D* (μm)	*f* (μm)	Intensity distribution *I*_*m*_	Transmittance function *t*_*i*_
**SOL #1**	20	1, 2	1: 1	FC363 53FEA 8F493 72F50 4B311
**SOL #2**	20	1, 2, 3	1: 1: 1	A1C25 2ECC1 57AC0 34BC7 374C9
**SOL #3**	20	1, 2, 3, 4, 5	1: 1: 1: 1: 1	7E233 D30EC D4BF8 807C4 C0AF1
**SOL #4**	20	1, 2, 3, 4	1: 1: 1: 1	FF763 735DE 490E8 32EED DDC0A
**SOL #5**	20	1, 2, 3, 4	0.4: 0.6: 0.8: 1	B79F5 8918D B64D9 C5C3F DADB1
**SOL #6**	20	1, 2, 4, 5	0.5: 1: 1: 0.5	DCC63 82C85 481DA ADC57 8FC50

**Figure 3:**
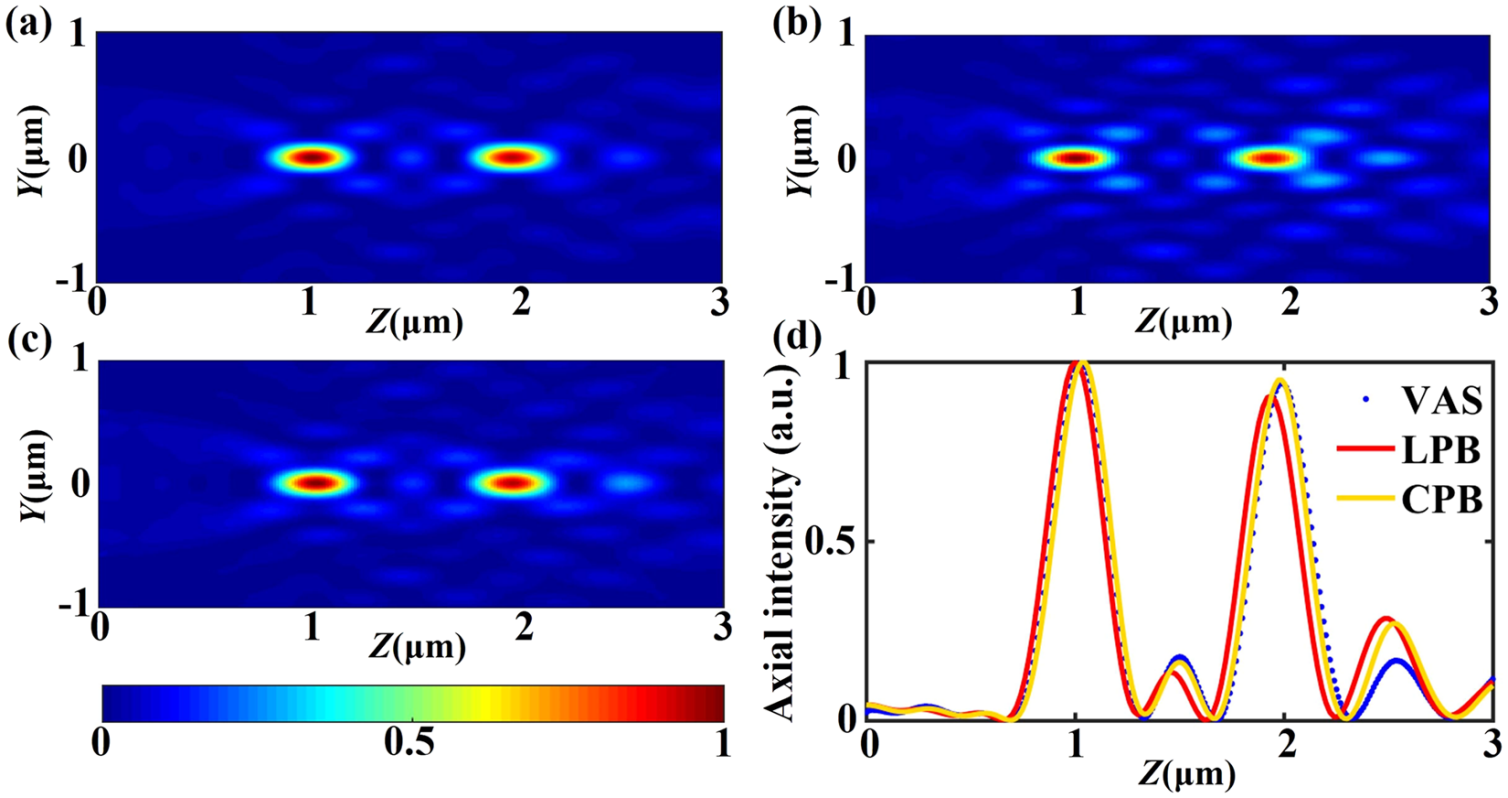
(**a**) The calculated electric-field pattern based on the VAS theory. (**b–c**) The simulated electric-field patterns at the *Y-Z* plane with LPB and CPB illumination, respectively. (**d**) Comparison of the axial intensity distributions for SOL #1 calculated via the VAS theory (blue dot), FDTD method with LPB (red solid line) and FDTD method with CPB (yellow solid line), respectively.

Firstly, we consider the design of a SOL that produces two focal spots with the equal intensity distribution and spacing between the spots on the *Z* axis under the illumination of a uniform plane wave. The two focal spots are located at 1 μm and 2 μm away from the output plane of SOL along the *Z* axis. A random initial transmittance function is used at the beginning of iteration. Through the method we mentioned before, a convergent solution that matches the requirements can be obtained. The axial intensity distribution of the diffractive pattern generated by the designed SOL #1 is displayed in Fig. 3. The normalized intensity distributions calculated by the VAS theory and FDTD are compared in the *Y-Z* plane. The calculated intensity distribution is shown in Fig. 3(a) together with the results of LPB simulation in Fig. 3(b) and CPB simulation in Fig. 3(c). The axial intensity distributions are further compared in Fig. 3(d). It can be seen that the electromagnetic simulation results are consistent with the VAS predictions especially for the main lobe of the focus. It can be clearly seen that the two sharp peaks emerge from the low background in the axial intensity distribution, and two sharp foci located at the designed positions are clearly visible. The interval between the adjacent focal spots is about 1 μm, as expected. There observes a good focusing effect for both the LPB and CPB incident lights, which implies a partly polarization-independence of the designed SOLs. For the SOLs illuminated by the radially or azimuthally polarized beam, focusing properties become different. Nonetheless, our method as described is applicable for arbitrary polarized waves, like the radially and azimuthally polarized beams, as long as the definition of optical field in Equation (1) is correspondingly modified.

**Figure 4:**
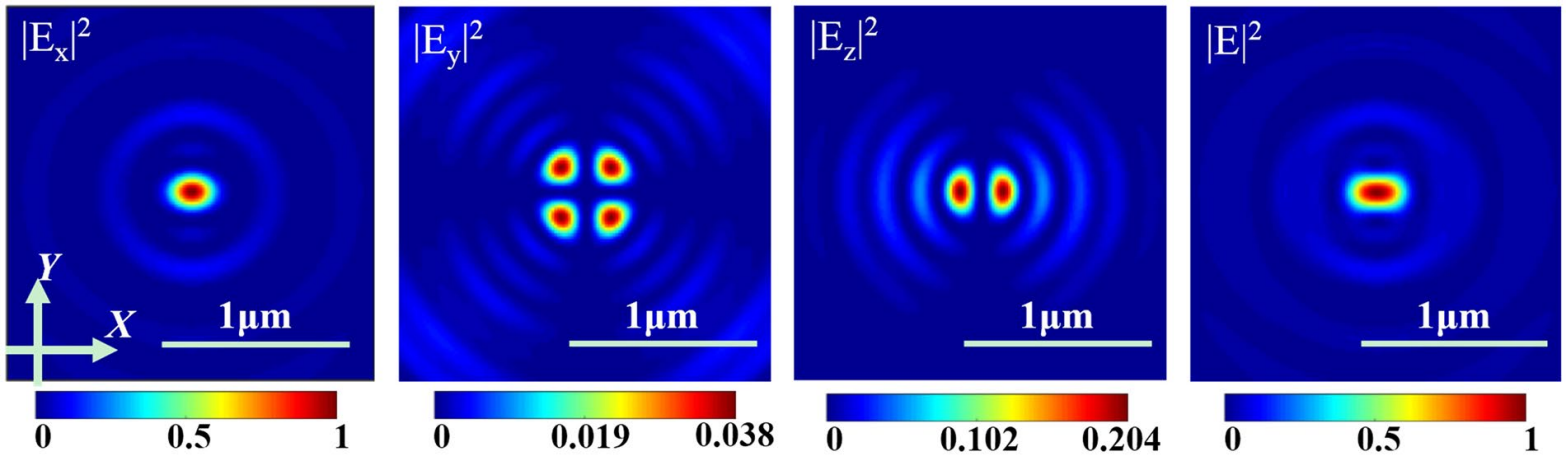
Normalized electric-field patterns for the first focus of SOL #1 achieved by the FDTD simulation.

According to the FDTD simulation results, for a linearly polarized plane wave (as an incident source), when the electric field polarizes along the *X* direction, we can see that in contrast to our VAS calculation, there exists the *|E*_*y*_ *|* ^2^ component, which exhibits a weak four-lobe intensity. Additionally, the longitudinal field component *|E*_*z*_ *|* ^2^ reveals an obvious two-lobe intensity pattern, as shown in Fig. 4. We usually ignore the slight *|E*_*y*_ *|* ^2^ component, as well as the longitudinal component which is difficult to measure^21, 22^. Thus, *|E*_*x*_ *|* ^2^ is used and successfully predicts the positions and appropriate sizes of the achieved foci.

**Figure 5:**
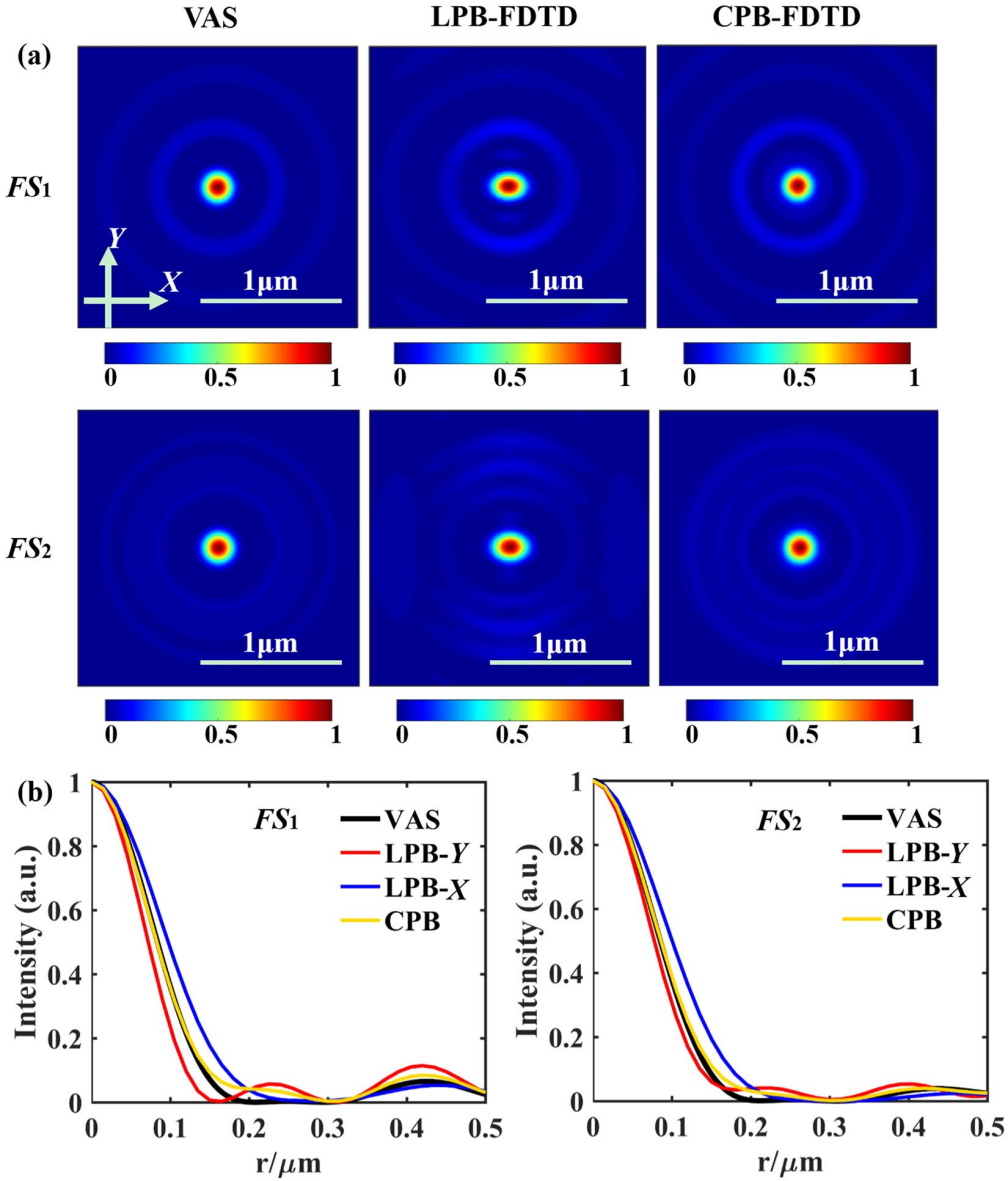
(**a**) Comparison of the electric-field components (*E*_*r*_) in the transverse focal planes behind the SOL #1. (**b**) The derived intensity profiles across the focal spots, for focal spot 1(*FS*_*1*_) and focal spot 2 (*FS*_*2*_), respectively.

The normalized intensity distributions in the transverse focal plane are compared in Fig. 5, which shows that the simulated focal planes agree well with the VAS calculation. However, the component |*E*_*x*_|^2^ of LPB calculated by FDTD is not rotationally symmetric as shown in Fig. 5, it is wider in the *X* direction than that in the *Y* direction, which can be explained by the more accurate and generalized VAS methods^27–29^. The FWHMs of all the focal spots along the *Y* axis are listed in Table 2, which are all beyond the calculated diffraction limit 0.61*λ*/NA (0.61*λ*/NA_1_ = *λ*/2.471, 0.61*λ*/NA_2_ = *λ*/2.435); NA_1_ and NA_2_ represent the numerical aperture of the focal spot 1 (*FS*_1_) and focal spot two (*FS*_2_), respectively, in the focal planes.

**Table 2:** Comparison of the designed and simulated results of the focal length *f* and FWHM for LPB and CPB.

	*f* (μm)				FWHM			
	VAS		FDTD		VAS		FDTD	
	LPB	CPB	LPB	CPB	LPB	CPB	LPB	CPB
***f***_**1**_ **= 1 μm**	1.03		1.00	1.04	*λ*/3.183		*λ*/3.696	*λ*/3.215
***f***_**2**_ **= 2 μm**	1.98		1.94	1.99	*λ*/3.115		*λ*/3.425	*λ*/3.087

**Figure 6:**
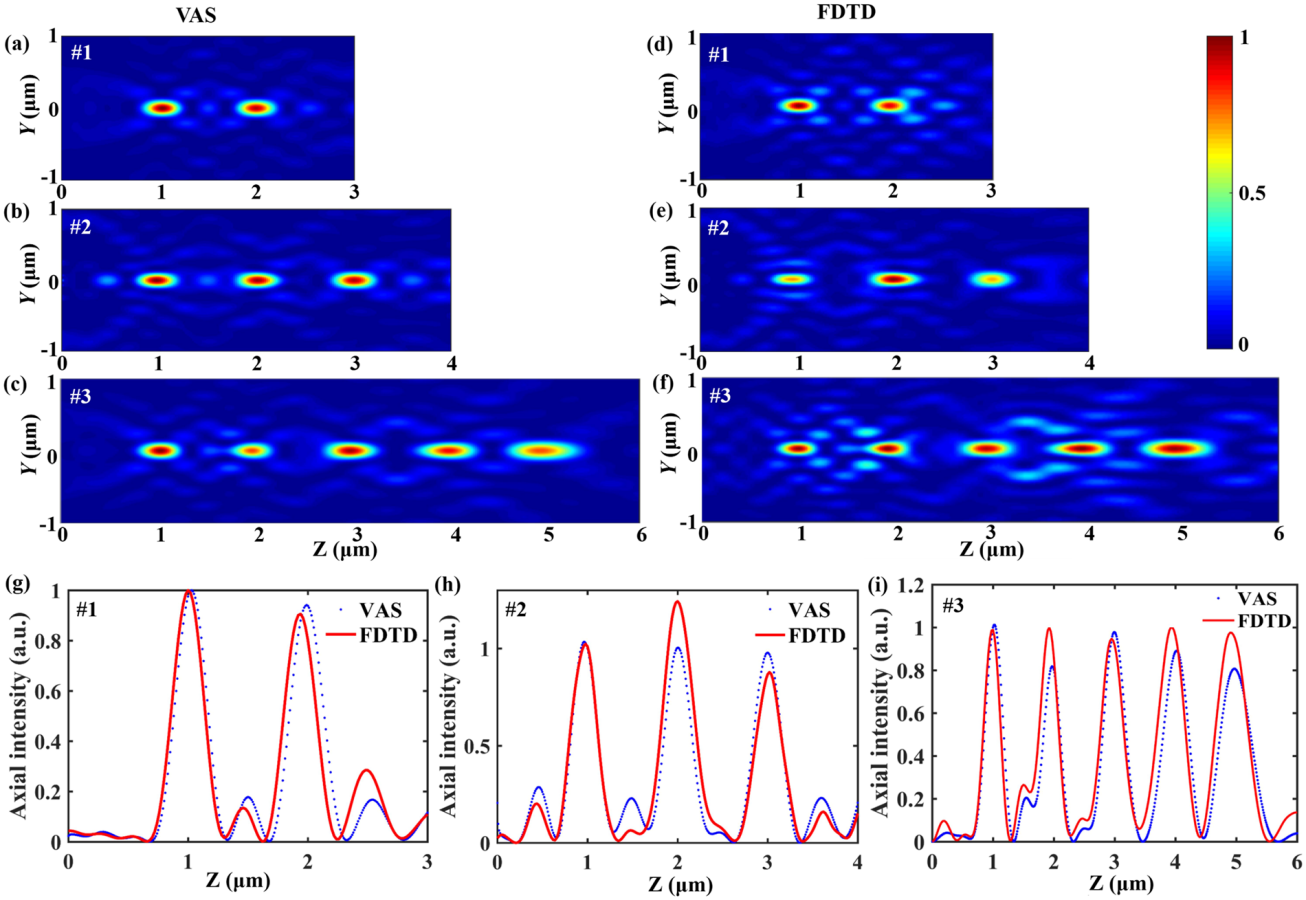
Comparison of the axial intensity distributions illuminated by LPB. (**a–c**) Show the normalized light intensity patterns of SOLs #1~#3 calculated by the VAS theory, respectively. (**d–f**) Show the normalized light intensity patterns of SOLs #1~#3 simulated by FDTD, respectively. (**g–i**) Show the comparison of the axial intensity distributions for SOLs #1~#3 calculated through the VAS theory (blue dot) and FDTD method (red solid line), respectively.

In order to show the flexible control over the light field with the optimization model, M is tuned to generate three or more spots along the optical axis. The intensity distribution of SOLs #1~#3 in the *Y-Z* plane is shown in Fig. 6 for the LPB illumination. For all the three SOLs, the intensities of the foci calculated by the VAS theory are almost the same, as demonstrated in Fig. 6(a)–(c); all the foci are strongly and exactly focused at the preset positions along the optical axis; meanwhile, the on-axis intensity distribution predicted by the VAS theory coincides with the rigorous electromagnetic simulation result by FDTD for these three SOLs. Comparing the focusing characteristics of SOLs #1~#3, the foci number shifts from 2 (for SOL #1) to 5 (for SOL #3). As shown in Fig. 6(d)–(f), the FWHMs of all the foci are relatively constant and beyond the calculated diffraction limit. For example, for the SOL #3 with the designed five focal spots, the simulated spot sizes at *f* = 1, 2, 3, 4 and 5 μm are *λ*/3.732, *λ*/3.481, *λ*/3.180, *λ*/3.465 and *λ*/2.878, respectively, compared to the calculated diffraction limit of *λ*/2.471, *λ*/2.435, *λ*/2.379, *λ*/2.306 and *λ*/2.221, respectively.

**Figure 7:**
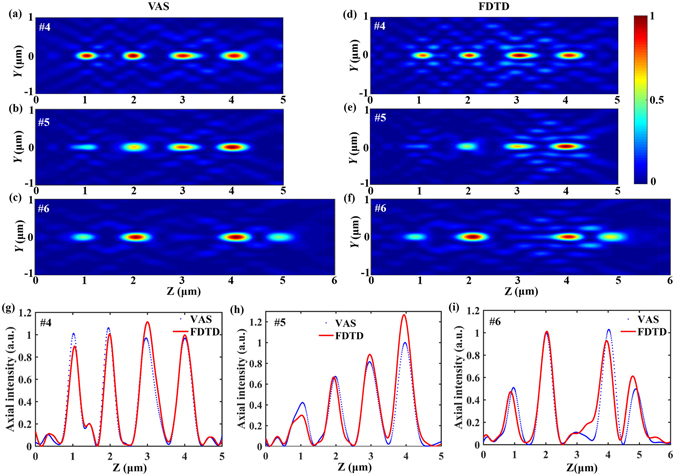
Comparison of the axial intensity distributions illuminated by LPB. (**a–c**) Show the normalized light intensity patterns of SOLs #4~#6 calculated by the VAS theory, respectively. (**d–f**) Show the normalized light intensity patterns of SOLs #4~#6 simulated by FDTD, respectively. (**g–i**) Show the comparison of the axial intensity distributions for SOLs #4~#6 calculated through the VAS theory (blue dot) and FDTD method (red solid line), respectively.

For the sake of practical applications, the ratio of the focusing intensity can also be adjusted. Through changing the parameter *μ* in the second objective function *Min*.(*I*_*2m*_) of the optimization model, we can modulate the intensity ratio of focal spots effectively. As shown in Fig. 7, the intensity distributions of the calculation and simulation results in the *Y-Z* plane demonstrate that the designed SOLs with the intensity ratios of 0.4:0.6:0.8:1 and 0.5:1:1:0.5 have been realized. Figure 7(b) and (c) show the generation of four focal spots with both different intensity distribution and separation. We measured the FWHMs of all the focal spots along the optical axis; for the SOL #5 with the designed focal length at *f* = 1, 2, 3 and 4 μm, the spot sizes are *λ*/4.289, *λ*/3.091, *λ*/3.897 and *λ*/3.706, respectively, all beyond the calculated diffraction limit, i.e. *λ*/2.471, *λ*/2.435, *λ*/2.379 and *λ*/2.306, respectively. Compared to the single-focusing SOLs, the focusing precision of the designed four-foci SOLs has some deviations from the preset focal lengths and distribution, which is related to the interference between the closely spaced focusing spots.

It should be noted that the coordinates of focal points can be chosen arbitrarily, and the light intensity patterns between the foci can be predetermined through the proposed method. The optimization model presented in this paper can be applied for the generation of any desired longitudinal intensity distribution.

## Conclusions

To sum up, we have shown an effective procedure for designing multifocal binary amplitude-type SOLs based on the VAS theory under the normal illumination of LPB or CPB. A GA optimization model has been proposed to control the focal spots’ properties and accelerate the computational process with the fast Hankel transform algorithm. Several focal distributions have been built. Meanwhile, a comparison of the VAS theoretical calculations and the FDTD simulation results has been made to confirm the optimization model. The simulated results show that all the designed SOLs by our method agree well with the desired expectations and have good focusing characteristics. Hotspots generated by SOLs #1~#6 show the resolution beyond the diffraction limit. Additionally, the focusing intensity of each focal spot can be tuned easily by changing the parameters of the optimization model. The optimization design introduced here is an effective and universal procedure, which can be extended to study the diffraction of different light contours with different vector beams by a binary amplitude-type SOL, such as light tunnels, doughnut-shape focal pattern, optical needle, and so on. Various peculiar focusing patterns may find important applications in optical trapping, particle acceleration, three-dimensional imaging and fluorescence microscopy.
